# Up-regulation of brain-expressed X-linked 2 is critical for hepatitis B virus X protein-induced hepatocellular carcinoma development

**DOI:** 10.18632/oncotarget.19477

**Published:** 2017-07-22

**Authors:** Fuqiang Huang, Pei Cai, Yanan Wang, Xian Zhou, Hongyu Chen, Wenjun Liao, Yilei Mao, Xiaojun Zha, Hongbing Zhang, Zhongdong Hu

**Affiliations:** ^1^ Modern Research Center for Traditional Chinese Medicine, School of Chinese Materia Medica, Beijing University of Chinese Medicine, Beijing, China; ^2^ State Key Laboratory of Medical Molecular Biology, Department of Physiology, Institute of Basic Medical Sciences and School of Basic Medicine, Graduate School of Peking Union Medical College, Chinese Academy of Medical Sciences and Peking Union Medical College, Beijing, China; ^3^ Department of Liver Surgery, Peking Union Medical College Hospital, Chinese Academy of Medical Sciences and Peking Union Medical College, Beijing, China; ^4^ Department of Biochemistry & Molecular Biology, School of Basic Medicine, Anhui Medical University, Hefei, China

**Keywords:** HCC, HBV, HBx, BEX2, osteopontin

## Abstract

Hepatocellular carcinoma (HCC) is the third leading cause of cancer-related death worldwide. Chronic hepatitis B virus (HBV) infection is a major cause for HCC. Hepatitis B virus X (HBx), one of four proteins encoded by HBV genome, plays a vital role in the pathogenesis of HBV-induced HCC. However, the molecular mechanisms of HBx-triggered HCC remain largely undetermined. Here we revealed that the expression of Brain-expressed X-linked 2 (BEX2) and Osteopontin (OPN) were elevated in liver tissues of HBV transgenic mice and human HCC specimens. Moreover, a positive correlation between BEX2 and OPN was exhibited in samples from HCC patients with HBV infection. The protein levels of BEX2 and OPN were both higher in HBV-positive HCC specimens compared to that of HBV-negative HCC specimens. HBx potentiated OPN expression through up-regulation of BEX2. Importantly, the depletion of BEX2 suppressed tumorigenic potential of HCC cells with highly expressed HBx. We demonstrated the important role of BEX2 in HCC pathogenesis, and BEX2 may be a novel therapeutic target for HCC patients with HBV infection. The newly identified HBx/BEX2/OPN signaling cassette is implicated in the pathogenesis of HBV-induced HCC.

## INTRODUCTION

Hepatocellular carcinoma (HCC) is one of the major malignancies and is the third leading cause of cancer-related death in the modern world [1, 2]. Chronic hepatitis B virus (HBV) infection is a dominant trigger for HCC [3]. More than 50% of the HCC cases are related to HBV infection worldwide [4]. The mechanisms whereby HBV infection induces HCC include the integration of HBV DNA into the host genome [5, 6], hepatic inflammation [7], and dysregulation of oncogenes or tumor suppressors [8, 9].

HBV genome encodes four proteins, including the polymerase (P), core protein (C), envelope protein (S), and X protein (HBx) [10, 11]. So far, there is evidence available supporting a pathogenetic role for HBx in HCC development [12-15]. HBx interacts with the transcription factors in the nucleus and modulates signaling pathways, thereby triggering hepatocyte transformation and uncontrolled proliferation [16-18]. It has been reported that HBx transcriptionally activates NF-κB through up-regulation of TBK1 [19]. Mitogen-activated protein kinases (MAPKs) are positively regulated by HBx [20]. In addition, HBx downregulates the expression of PTEN by functionally inhibiting the activity of tumor suppressor P53 [9]. Previous studies have reported that HBx positively regulated Osteopontin (OPN) expression in HCC cells [21, 22]. OPN, a secreted glycoprotein, is expressed widely and positively correlated with tumorigenesis of multiple cancers, including HCC [23, 24]. OPN is regarded as a novel marker for early HCC [25]. Elevated OPN expression is closely associated with early recurrence and poor prognosis of HCC [26].

Brain-expressed X-linked 2 (BEX2) is one of BEX family members and plays an important function in the development of nervous system [27]. Recently, the role of BEX2 in cancer is characterized in numerous studies. BEX2 promotes the growth of breast cancer cells partly through up-regulation of NF-κB signaling [28]. BEX2 is also involved in the development of glioma [29]. Additionally, our previous study has reported that BEX2 was essential to the tumorigenesis of cells with activated mTOR [30]. However, the role of BEX2 in the development of HBV-associated HCC remains unknown.

In this study, we found the elevated expression of BEX2 and OPN in liver tissues of HBV transgenic mice and human HCC specimens, and there was a potential positive correlation between BEX2 and OPN in HCC. Moreover, HBx up-regulation of OPN was mediated by BEX2. Importantly, BEX2 was critical for the growth of HCC cells with highly expressed HBx *in vitro* and *in vivo*. Therefore, this newly discovered mechanism contributed to unveil the molecular genesis of HCC. Targeting BEX2 may be a promising strategy for treatment of HBV-induced HCC.

## RESULTS

### The expression of BEX2 and OPN is elevated in liver tissues of HBV transgenic mice

**Figure 1:**
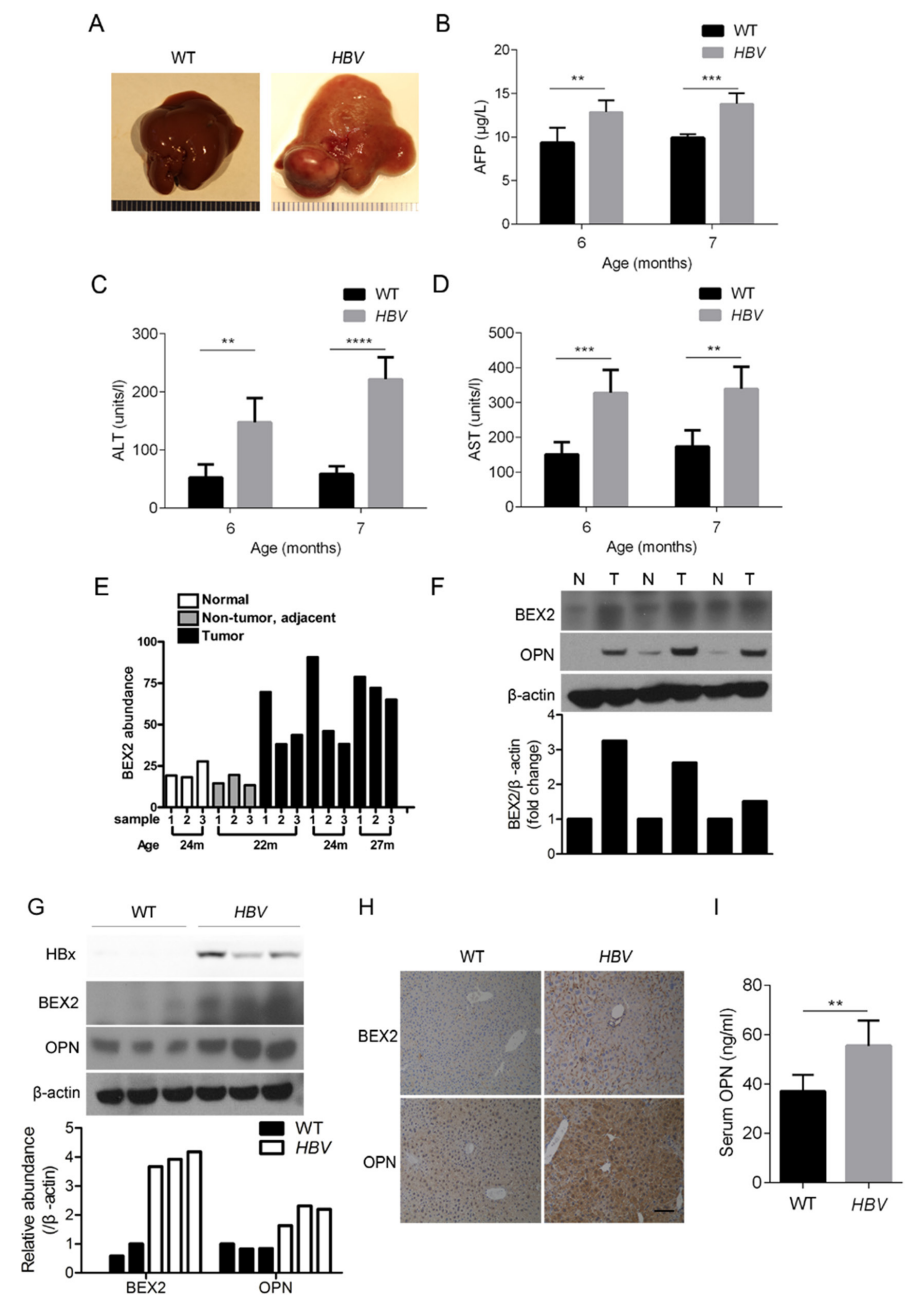
BEX2 and OPN are increased in liver tissues of HBV transgenic mice **(A)** Macroscopic appearance of livers from 14-month-old wide-type (WT) and HBV transgenic mice. **(B-D)** The levels of AFP (B), ALT (C), and AST (D) in the serum from WT and HBV transgenic mice at 6 months and 7 months (n=5). **P < 0.01, ***P < 0.001, ****P < 0.0001. **(E)** Analysis of the mRNA abundance of BEX2 in liver tissues from HCC mouse model by using mRNA microarray data from the Gene Expression Omnibus database (GEO accession number GSE2127). m: month. **(F)** Liver tumor tissues (T) and the adjacent liver tissues (N) from 14-month-old HBV transgenic mice were lysed and then subjected to immunoblotting (upper panel). The relative quantitation of BEX2 by scanning densitometry analysis upon normalization for β-actin (lower panel). **(G)** Liver tissues dissected from 4-month-old WT and HBV transgenic mice were lysed and then subjected to immunoblotting (upper panel). The relative quantitation of BEX2 and OPN by scanning densitometry analysis upon normalization for β-actin (lower panel). **(H)** Immunohistochemical analysis of liver tissues from 4-month-old WT and HBV transgenic mice with antibodies against BEX2 and OPN. Representative images were presented. Scale bar, 100 μm. **(I)** ELISA analysis of OPN levels in the serum from 4-month-old WT and HBV transgenic mice (n=6). **P < 0.01.

HBV transgenic mouse model was used to explore the potential molecular events in the development of HBV-induced HCC. In line with the previous study, liver tumors were observed in HBV transgenic mice at the age of 14 months (Figure 1A). α-fetoprotein (AFP), a serum marker of HCC, was increased in the serum of HBV mice compared with that of wide-type mice (Figure 1B). Moreover, the elevated levels of both ALT and AST suggested that severe liver injury occurred in HBV mice (Figure 1C and 1D). Through data mining in the Gene Expression Omnibus database (GEO accession number GSE2127, deposited by Lusis AJ) [31], we found that the abundance of BEX2 was higher in the liver tumor tissues, in comparison to the normal and paratumor tissues in a mouse model of HCC (Figure 1E). This data implicated BEX2 in the pathogenesis of HCC. To determine the role of BEX2 in HCC, we first examined the expression of BEX2 in the liver tumor tissues of HBV mice. The level of BEX2 was higher in the liver tumor tissues than in the adjacent normal tissues of HBV mice (Figure 1F). Furthermore, BEX2 expression was increased in the livers of HBV mice compared with that of wide-type mice (Figure 1G). Immunohistochemical analysis also showed that BEX2 expression was elevated in the liver tissues of HBV mice (Figure 1H). Osteopontin (OPN), a secreted glycoprotein, is highly expressed in HCC and is reported as a new marker of the early stage of HCC [32]. OPN abundance was dramatically increased in the tumor tissues compared to the paratumor tissues of HBV mice (Figure 1F). Moreover, the expression of OPN was upregulated in the liver tissues of HBV mice (Figure 1G and 1H). We also found that the serum OPN levels of HBV mice were increased compared to that of wide-type mice (Figure 1I).

### Enhanced expression of BEX2 and OPN in human HCC specimens

**Figure 2:**
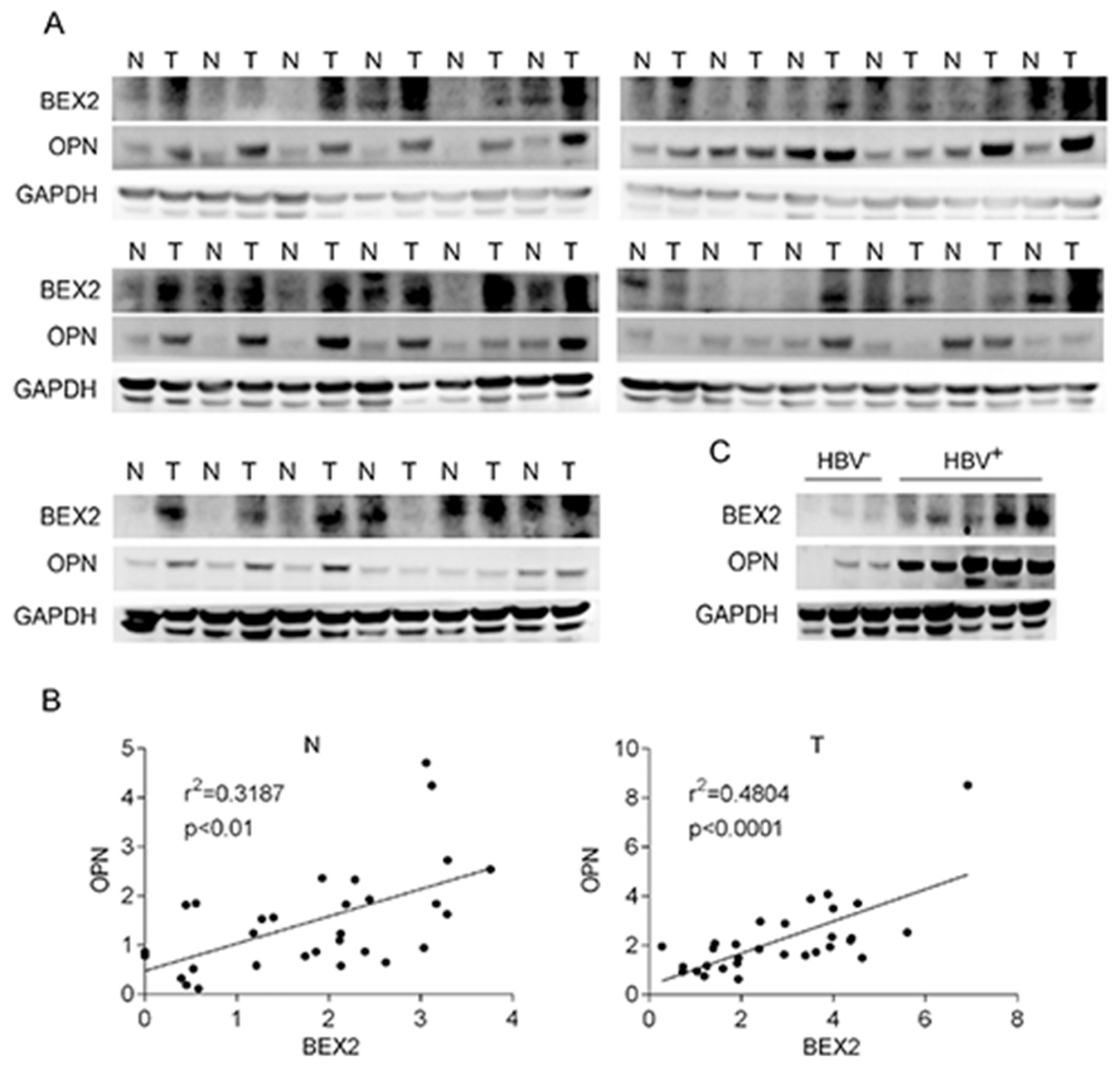
Enhanced expression of BEX2 and OPN in human HCC specimens **(A)** Liver tumors (T) and the adjacent tissues (N) from 30 HCC patients were subjected to immunoblotting for analysis of BEX2 and OPN expression. **(B)** Statistical analysis of the correlation between BEX2 and OPN in human HCC specimens. **(C)** HBV-negative (HBV^-^) and HBV-positive (HBV^+^) HCC specimens were subjected to immunoblotting for analysis of BEX2 and OPN expression.

To extend our findings to clinical relevance, the expression of BEX2 and OPN was checked in 30 pairs of specimens from HCC patients. We found that BEX2 and OPN were elevated in 70% (21/30) and 73% (22/30) HCC specimens in comparison with their adjacent normal tissues, respectively (Figure 2A). Furthermore, OPN abundance was increased in 81% (17/21) HCC specimens with elevated BEX2, and BEX2 was increased in 86% (19/22) HCC specimens with elevated OPN (Figure 2A). As shown in Figure 2B, there was a significant correlation between BEX2 and OPN in HCC. In addition, both BEX2 and OPN expression were increased in HBV-positive HCC specimens compared with HBV-negative HCC specimens (Figure 2C).

### HBx potentiates OPN expression through up-regulation of BEX2

**Figure 3:**
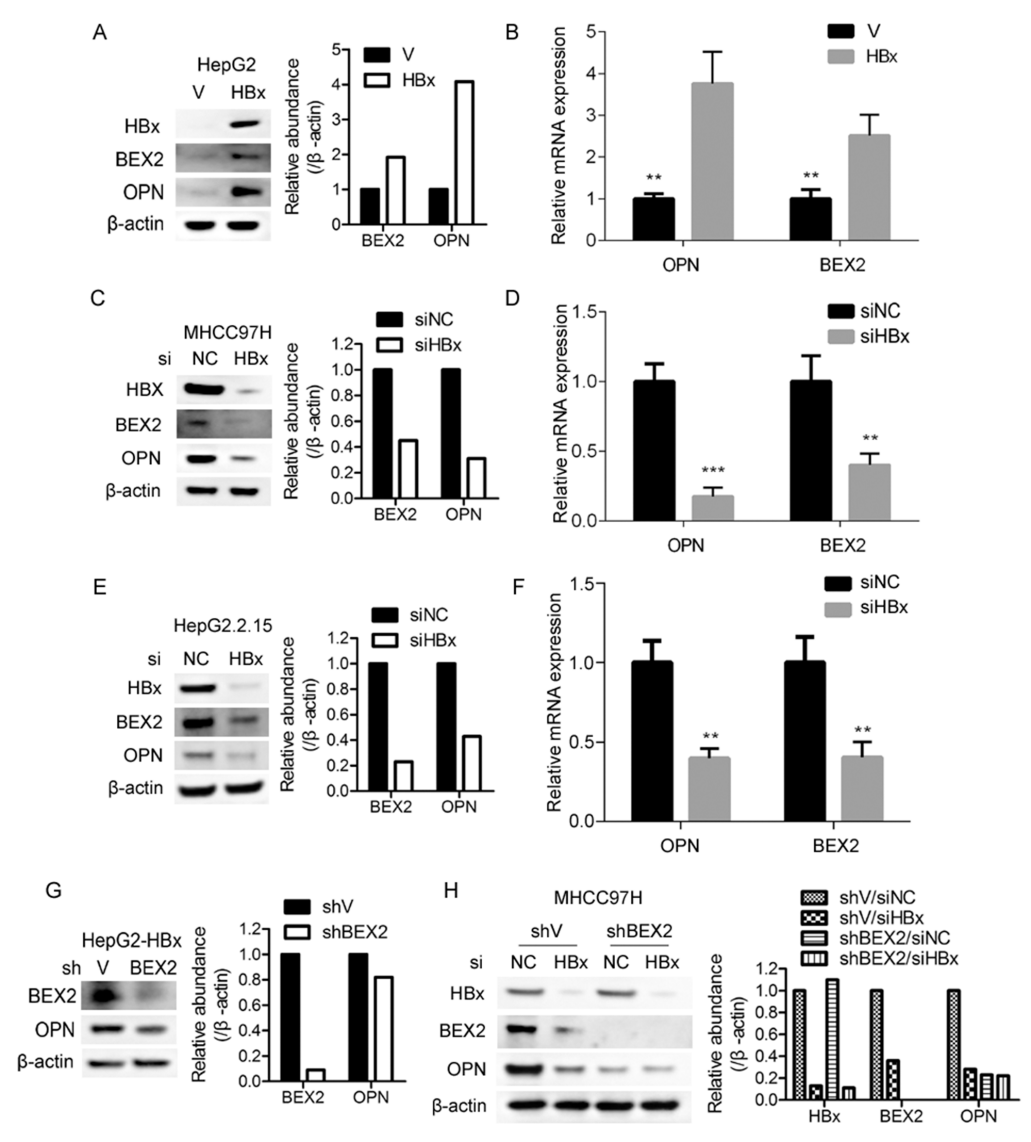
HBx enhances OPN expression via up-regulation of BEX2 **(A)** and **(B)** Cell lysates and total RNA extracted from HepG2 cells overexpressing HBx or the control vector were subjected to immunoblotting and qRT-PCR, respectively. **P < 0.01. **(C)** and **(D)** Cell lysates and total RNA extracted from MHCC97H cells transfected with siRNAs targeting HBx or negative control were subjected to immunoblotting and qRT-PCR, respectively. **P < 0.01, ***P < 0.001. **(E)** and **(F)** Cell lysates and total RNA extracted from HepG2.2.15 cells transfected with siRNAs targeting HBx or negative control were subjected to immunoblotting and qRT-PCR, respectively. **P < 0.01. **(G)** HBx-overexpressed HepG2 cells infected with shBEX2 or scramble shRNA-expressing lentiviruses were subjected to immunoblotting. **(H)** shBEX2 or shV stably expressed MHCC97H cells transfected with siRNAs targeting HBx or negative control were subjected to immunoblotting.

To investigate whether there is a putative regulatory relationship among HBx, BEX2, and OPN, firstly HBx was overexpressed in HepG2 cells. Ectopic expression of HBx dramatically augmented the protein levels of BEX2 and OPN (Figure 3A). qRT-PCR analysis revealed that the mRNA of BEX2 and OPN were also increased by overexpressed HBx in HepG2 cells (Figure 3B). Moreover, depletion of HBx with RNA interference markedly decreased the protein and mRNA of BEX2 and OPN in MHCC97H and HepG2.2.15 cells (Figure 3C-3F). Furthermore, knockdown of BEX2 suppressed OPN expression in HBx-overexpressed HepG2 cells (Figure 3G). In addition, we investigated whether BEX2 was involved in HBx up-regulation of OPN by knocking down HBx in shBEX2 or shV stably expressed MHCC97H cells. As depicted in Figure 3H, depletion of HBx dramatically suppressed OPN level in shV stably expressed MHCC97H cells, whereas knockdown of HBx had no effect on OPN expression in MHCC97H cells with reduced BEX2. Collectively, HBx upregulates BEX2/OPN signaling cascade in HCC cells.

### Depletion of BEX2 blunts the proliferation of HCC cells with highly expressed HBx

**Figure 4:**
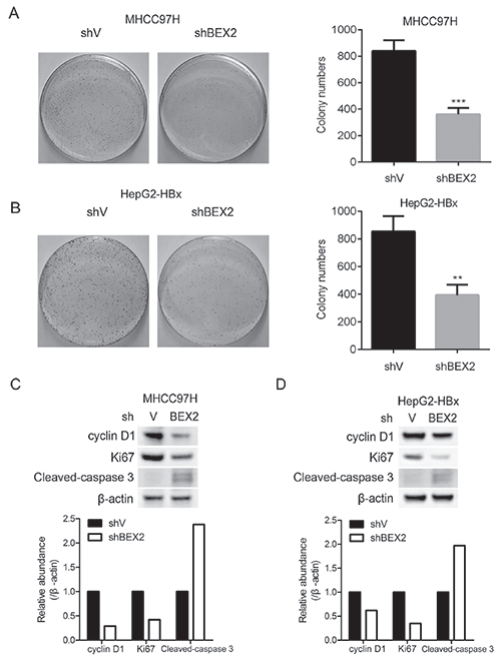
Depletion of BEX2 inhibits the proliferation of HCC cells with highly expressed HBx **(A)** and **(B)** MHCC97H cells or HBx-overexpressed HepG2 cells infected with shBEX2 or scramble shRNA-expressing lentiviruses were subjected to colony formation assay. (Left panel): Representative images; (Right panel): quantitative data. **P < 0.01, ***P < 0.001. **(C)** and **(D)** MHCC97H cells or HBx-overexpressed HepG2 cells infected with shBEX2 or scramble shRNA-expressing lentiviruses were subjected to immunoblotting (upper panel). The relative quantitation of the indicated proteins by scanning densitometry analysis upon normalization for β-actin (lower panel).

To investigate the role of BEX2 in HBV-induced HCC, we silenced BEX2 in HCC cells and examined their colony formation. Knockdown of BEX2 significantly inhibited the colony formation of MHCC97H cells (Figure 4A). Likewise, depletion of BEX2 markedly suppressed the colony formation of HepG2 cells with ectopically expressed HBx (Figure 4B). In addition, silencing BEX2 led to down-regulation of cyclin D1 (a key regulator of cell cycle) and Ki-67 (a proliferation marker), and up-regulation of cleaved-caspase 3 (an apoptosis marker) in MHCC97H cells and HBx-overexpressed HepG2 cells (Figure 4C and 4D). Therefore, blockage of BEX2 inhibited the growth of HCC cells partially through induction of cell cycle arrest and activation of apoptosis.

### Reduction of BEX2 restrains the tumorigenesis of HCC cells

**Figure 5:**
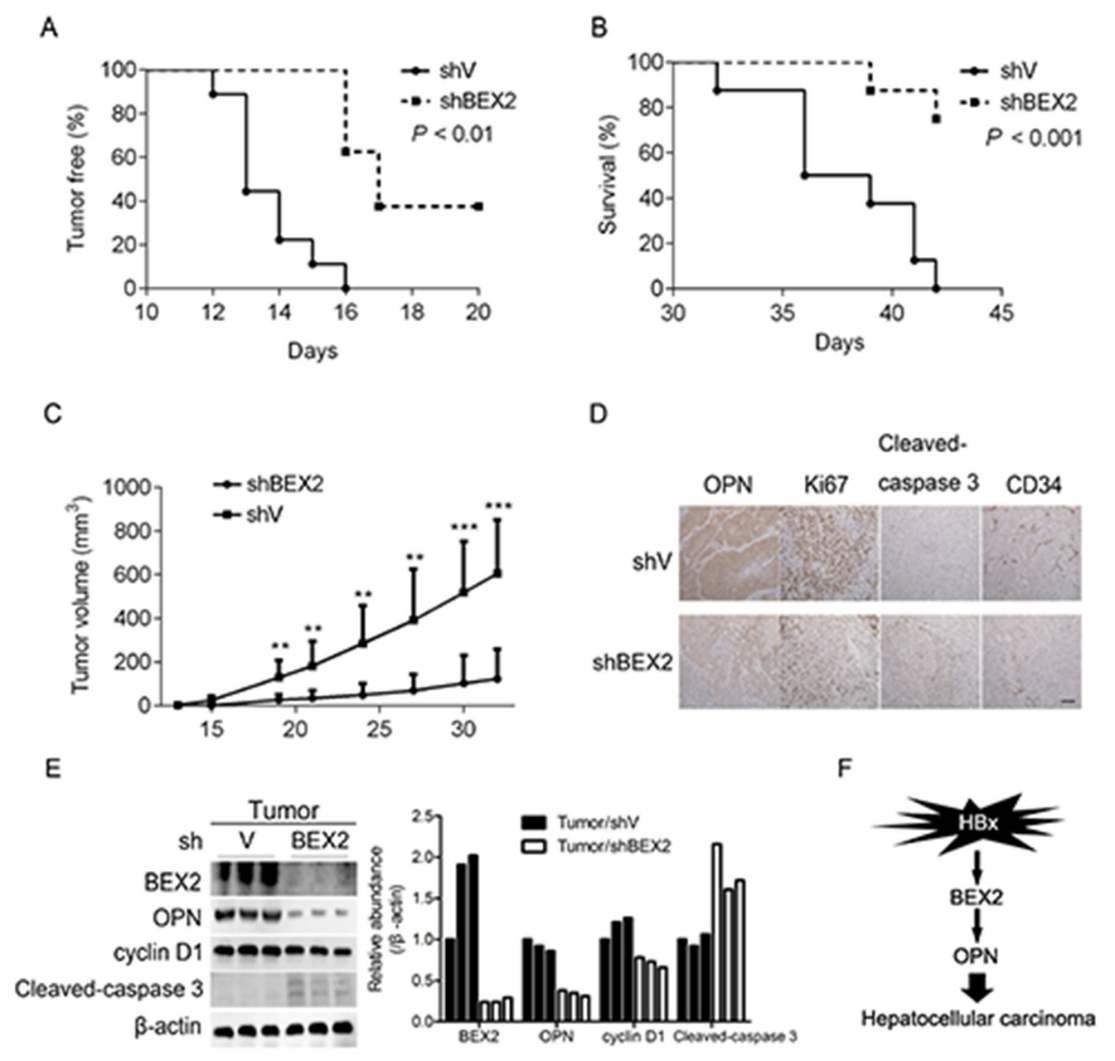
Reduction of BEX2 suppresses the tumorigenicity of HCC cells **(A-C)** MHCC97H cells transduced with shV or shBEX2 lentiviruses were inoculated subcutaneously into nude mice, and followed for tumor initiation (A), mice survival (B), and tumor volume (C). **P < 0.01, ***P < 0.001. **(D)** Tumor tissues from nude mice were subjected to immunohistochemical analysis of OPN, Ki67, cleaved-caspase 3, and CD34. Representative images were shown. Scale bar, 100 μm. **(E)** Tumor tissues from nude mice were lysed and then subjected to immunoblotting (left panel). The relative quantitation of the indicated proteins by scanning densitometry analysis upon normalization for β-actin (right panel). **(F)** Schematic illustration of HBx up-regulation of OPN via BEX2, thus promoting HCC development.

To explore the *in vivo* role of BEX2 in the oncogenesis of HCC cells, the tumorigenic capacity of MHCC97H cells harboring shRNA against negative control or BEX2 was evaluated in a nude mice model. Depletion of BEX2 significantly retarded the tumorigenicity of MHCC97H cells, as indicated by tumor initiation, survival, and tumor volume (Figure 5A-5C). Moreover, IHC analysis revealed that MHCC97H cells with knockdown of BEX2 exhibited remarkably decreased expression of OPN, reduction of proliferation and angiogenesis, as well as enhanced apoptosis *in vivo* (Figure 5D). In addition, immunoblot analysis showed that reduction of BEX2 led to decreased expression of OPN and cyclin D1, and an increase in the level of cleaved-caspase 3 in tumor tissues derived from MHCC97H cells (Figure 5E). Collectively, BEX2 is required for the tumorigenesis of HCC cells with highly expressed HBx.

## DISCUSSION

Chronic HBV infection is the most common cause of HCC worldwide [4]. However, the mechanisms responsible for the development of HBV-induced HCC remain largely unknown. There has been much evidence suggesting that HBx, one of four proteins encoded by HBV genome, plays a critical role in the pathogenesis of HBV-related HCC through influencing several cellular processes including cell cycle progression [33], apoptosis [34], DNA repair [35], protein degradation [36], and signal transduction [16]. However, the underlying mechanisms of HBx-induced HCC remain obscured. Here we found that there was a positive correlation between BEX2 and OPN in HCC samples which were both increased in liver tissues of HBV transgenic mice and human HCC specimens. Furthermore, HBx enhanced OPN expression via up-regulation of BEX2 in HCC cells. In addition, the depletion of BEX2 significantly suppressed the tumorigenic capacity of HCC cells with highly expressed HBx.

HBV transgenic mouse is an ideal model widely used for defining the molecular mechanisms responsible for HBV-associated HCC [37, 38]. Consistent with the previous study, we found that HCC was developed in HBV transgenic mouse, and AFP, ALT, and AST were all elevated in the serum of HBV mice. BEX2 is overexpressed in breast cancer and facilitates the growth of breast cancer cells [28, 39]. It is also reported that BEX2 expression is upregulated in glioma tissues [40]. Additionally, BEX2 expression was increased in MLL mutant acute myeloid leukemia (AML) cells [41]. Herein we demonstrated that BEX2 expression was dramatically increased in liver tumor tissues of HBV transgenic mice. BEX2 was also elevated in liver tumor tissues in comparison with adjacent liver tissues in the most of human HBV-related HCC specimens examined. Moreover, BEX2 was increased in HBV-related HCC specimens compared with HBV-negative HCC specimens. Thus, we proposed that BEX2 with an oncogenic role may be implicated in the pathogenesis of HBV-associated HCC. In addition, we demonstrated that blockade of BEX2 prominently inhibited the colony formation and tumorigenicity of HCC cells with highly expressed HBx. Taken together, BEX2 is a promising target for treatment of HBV-induced HCC.

OPN is overexpressed in HCC and is a useful diagnostic marker for early HCC [25]. OPN was identified to be associated with metastasis of HCC [42]. However, the regulatory relationship between HBV and OPN in HCC has been poorly characterized. It was reported that HBx activated the promoter activity of OPN and upregulated OPN expression through 5-lipoxygenase in HCC cells [21, 22]. In this study, we demonstrated that OPN was indeed markedly upregulated in liver tumor tissues and serum of HBV transgenic mouse, as well as the majority of HCC specimens. OPN expression was also elevated in HBV-positive HCC specimens compared to HBV-negative HCC specimens. Furthermore, there was a potential positive correlation between BEX2 and OPN in HBV-related HCC. We also revealed that HBx augmented OPN expression through up-regulation of BEX2 in HCC cells. Collectively, up-regulation of BEX2/OPN cascade contributed to the development of HBx-induced HCC (Figure 5F). Targeting this newly identified HBx/BEX2/OPN pathway may be a promising therapeutic strategy for HBV-associated HCC.

In this study, HBx/BEX2/OPN signaling pathway is partially responsible for HCC development driven by HBV infection. BEX2 is uncovered as a new molecular effector of HCC and may be a novel candidate target for HCC treatment.

## MATERIALS AND METHODS

### Reagents and antibodies

FBS and DMEM were from HyClone (Logan, UT, USA). Lipofectamine 2000 and 4-12% Bis-Tris NuPAGE gels were from Invitrogen (Carlsbad, CA, USA). Antibodies against OPN, BEX2, HBx, Ki-67, and CD34 were from Abcam (Cambridge, MA, USA). Antibody against cleaved-caspase 3 was from Cell Signaling Technology (Danvers, MA, USA). Antibody against β-actin and all the secondary antibodies were from Santa Cruz Technology (Santa Cruz, CA, USA). Antibodies against cyclin D1 and GAPDH were from Abclonal (Cambridge, MA, USA).

### Cell culture

HepG2 cells were from American Type Culture Collection (Manassas, VA, USA). MHCC97H cells were from Liver Cancer Institute, Zhongshan Hospital, Fudan University, China. HepG2.2.15cells were kindly provided by Wei Wang (Peking University Hepatology Institute, Beijing, China). Cells were cultured in Dulbecco modified Eagle’s medium (DMEM) with 10% FBS and 1% penicillin/streptomycin in 5% CO_2_ at 37°C.

### Colony formation

A total of 6,000 cells were seeded in 100 mm dishes and cultured for 10 or 14 days, and then fixed with methanol for 15 min and stained with 0.1% crystal violet. Cell colonies stained were photographed and counted using photoshop CS6 software.

### Knockdown of BEX2 in MHCC97H cells

The shRNA sequence targeting human BEX2 was cloned into pLL3.7 vector through Hpa I and Xho I sites. The shRNA sequences were as follows:

BEX2 forward: 5’-TGGACATAATGCATAGGCTTTTCAAGAGAAAGCCTATGCATTATGTCCTTTTTTC-3’, BEX2 reverse: 5’-TCGAGAAAAAAGGACATAATGCATAGGCTTTCTCTTGAAAAGCCTATGCATTATGTCCA-3’. 293T cells were cotransfected with pLL3.7-shRNA and the packaging vectors ( VSVG, REV, and pMDL). Forty-eight hours later, supernatants were collected and filtered with a 0.45 μm filter for infection of MHCC97H cells. The infection rate was assessed according to the expression of green fluorescent protein after incubation with virus for 48 h.

### RNA interference

All siRNA oligonucleotides were synthesized from GenePharma (Shanghai, China). Cells were seeded in 6-well plates and transfected with indicated siRNAs according to the manufacturer’s protocol. The siRNA sequences were as follows: Negative Control (NC): 5’-UUCUCCGAACGUGUCACGUdTdT-3’; HBx: 5’-CCGACCUUGAGGCAUACUUdTdT-3’.

### Immunoblot analysis

Cells were washed with PBS and harvested with lysis buffer (2% SDS, 100 mM DTT, 10 mM Tris (pH 6.8), and 10% glycerol), then boiled for 10 min and centrifuged at 13,000 rpm for 5 min. The levels of the indicated proteins were detected by western blotting described previously [30]. The relative quantitation of immunoblotting results was performed using image J software.

### Quantitative real-time PCR

Total RNA was extracted from cells with TRIzol reagent (Invitrogen). One microgramRNA was subjected to reverse transcription reaction using ReverTra Ace® qPCR RT Master Mix (TOYOBO, Japan). Amplification was performed for 40 cycles using cDNA as the template with TransStart Green qPCR SuperMix (TransGen Biotech, Beijing, China) in a quantitative real time PCR reaction. TATA-binding protein (TBP) serves as an internal control. The primer sequences were as follows: OPN, forward: 5’-GAAGTTTCGCAGACCTGACAT-3’, and reverse: 5’-GTATGCACCATTCAACTCCTCG-3’; BEX2, forward: 5’-AAAGAGGAACGAGCGTTAAACA-3’, and reverse: 5’-TCACTAACATTCAAAGGTAGGGC-3’; TBP, forward: 5’-GAGCCAAGAGTGAAGAACAGTC-3’, and reverse: 5’-GCTCCCCACCATATTCTGAATCT-3’.

### Liver assessment of HBV transgenic mice

HBV transgenic mice (C57BL/6J-Tg (Alb1HBV) 44Bri/J) contain HBV genome S, pre-S, and X domains under the mouse albumin promoter. The mice spontaneously developed HCC at the age of 14 months [43]. The liver tissues were harvested for immunoblot and immunohistochemistry analysis. All the mice were maintained according to the Animal Care and Use Committee Guidelines of Peking Union Medical College.

### Biochemical analysis of mice serum

Serum collected from HBV transgenic and wide-type mice were subjected to biochemical analysis. The level of AFP was measured with Fetoprotein assay kit (Nanjing Jiancheng Bioengineering Institute, Nanjing, China). The levels of ALT and AST were examined in the department of laboratory medicine of Peking Union Medical College Hospital. The level of OPN was examined using Quantikine Mouse/Rat OPN ELISA kits (R&D Systems, Minneapolis, MN, USA) according to the manufacturer’s protocol [44].

### Human HCC sample assessment

Human HCC specimens were obtained from patients who underwent surgery at Peking Union Medical College Hospital. Tumor tissues were extracted with lysis buffer and then subjected to immunoblotting. All the patients provided written informed consent. All the procedures were performed under the permission of the Peking Union Medical College Hospital Ethics Board.

### Xenografting tumorigenesis

Immunodeficient nude mice (BALB/c, 4-5 weeks old) were obtained from Beijing Vital River Laboratory Animal Technology Co. Ltd. Eight male mice were in each cohort. A total of 4×10^5^ MHCC97H cells expressing shBEX2 or scramble shRNA in 100 μl of DMEM were subcutaneously inoculated into the right posterior back region of nude mice. The subcutaneous tumor model was established as described previously [45].

### Immunohistochemistry (IHC) analysis

Immunohistochemical staining was performed as described previously [46]. In brief, liver tissues or tumor tissues were fixed in 4% paraformaldehyde and embedded in paraffin for H&E staining and immunohistochemical analysis of indicated proteins.

### Statistical analysis

The Kaplan-Meier log-rank test was used for the analysis of tumor development and mice survival. The two-tailed Student’s t-test was used for the analysis of differences between two groups in GraphPad Prism software. Data represents the mean ± SEM of triplicate samples. Statistical significance was defined as *P*<0.05.

## Abbreviations

HCC: hepatocellular carcinoma
HBV: hepatitis B virus
HBx: hepatitis B virus X
BEX2: brain-expressed X-linked 2
OPN: osteopontin
AFP: α-fetoprotein
TBP: TATA-binding protein
IHC: immunohistochemistry
